# The Diseases of the Ear

**Published:** 1860-05

**Authors:** 


THE DISEASES OF THE EAR; their Nature, Diagnosis and Treatment. By JOSEPH TOYNBEE, F. R. S. With one hundred engravings. Blanchard & Lea, Philadelphia.

In the preparation of the above work, the author's aim has been to produce a practical treatise on the Diseases of the Ear. We learn that the labors and investigations that are made the basis of the present treatise, have affected more for Aural Pathology than those of all his predecessors either in England or on the Continent. From the list of "Published papers on the Structure, Functions, and Diseases of the Ear," given in the Appendix, we should infer that Mr. Toynbee has labored extensively and with effect, to discover and describe the morbid appearances that disease has produced in this important organ, and has presented an accumulation of facts that must lay the foundation for a more rational mode of treating the special diseases of this organ than has heretofore been resorted to.

The researches of the author substantiate the fact that the great majority of the diseases of the ear, resulting in deafness, have their origin in inflammation of one kind or another. And as a consequence, if the members of our profession would devote so much of their attention to this subject as its importance demands, there would be no necessity of making these affections a speciality; and the quack advertiser and humbugging specialist would no longer prey upon the unsuspecting and ignorant sufferer.

The above work is to be had at the Book Store of S. C. Griggs & Co., Chicago.

